# Pleozymes: Pleiotropic Oxidized Carbon Nanozymes Enhance Cellular Metabolic Flexibility

**DOI:** 10.3390/nano14242017

**Published:** 2024-12-15

**Authors:** Anh T. T. Vo, Karthik Mouli, Anton V. Liopo, Philip Lorenzi, Lin Tan, Bo Wei, Sara A. Martinez, Emily A. McHugh, James M. Tour, Uffaf Khan, Paul J. Derry, Thomas A. Kent

**Affiliations:** 1 Center for Genomics and Precision Medicine, Institute of Bioscience and Technology, Texas A&M Health Science Center, Houston, TX 77030, USA; anhvo0915@tamu.edu (A.T.T.V.); kmouli@tamu.edu (K.M.); liopo@tamu.edu (A.V.L.); uk2015@tamu.edu (U.K.); 2 Department of Chemistry, Rice University, Houston, TX 77005, USA; emily.mchugh@utexas.edu (E.A.M.); tour@rice.edu (J.M.T.); 3 Metabolomics Core Facility, Department of Bioinformatics & Computational Biology, The University of Texas MD Anderson Cancer Center, Houston, TX 77030, USA; pllorenzi@mdanderson.org (P.L.); ltan@mdanderson.org (L.T.); bwei1@mdanderson.org (B.W.); samartinez3@mdanderson.org (S.A.M.); 4 Smalley-Curl Institute, Rice University, Houston, TX 77005, USA; 5 Rice Advanced Materials Institute, Rice University, Houston, TX 77005, USA; 6 The NanoCarbon Center, Rice University, Houston, TX 77005, USA; 7 School of Engineering Medicine, Texas A&M University, Houston, TX 77030, USA; 8 Stanley H. Appel Department of Neurology, Houston Methodist Hospital and Houston Methodist Research Institute, Houston, TX 77030, USA

**Keywords:** nanozyme, mitochondrial metabolism, NADH, fatty acid oxidation, oxidized activated carbon nanoparticles, redox mediator

## Abstract

Our group has synthesized a pleiotropic synthetic nanozyme redox mediator we term a “pleozyme” that displays multiple enzymatic characteristics, including acting as a superoxide dismutase mimetic, oxidizing NADH to NAD^+^, and oxidizing H_2_S to polysulfides and thiosulfate. Benefits have been seen in acute and chronic neurological disease models. The molecule is sourced from coconut-derived activated charcoal that has undergone harsh oxidization with fuming nitric acid, which alters the structure and chemical characteristics, yielding 3–8 nm discs with broad redox potential. Prior work showed pleozymes localize to mitochondria and increase oxidative phosphorylation and glycolysis. Here, we measured cellular NAD^+^ and NADH levels after pleozyme treatment and observed increased total cellular NADH levels but not total NAD^+^ levels. A ^13^C-glucose metabolic flux analysis suggested pleozymes stimulate the generation of pyruvate and lactate glycolytically and from the tricarboxylic acid (TCA) cycle, pointing to malate decarboxylation. Analysis of intracellular fatty acid abundances suggests pleozymes increased fatty acid β-oxidation, with a concomitant increase in succinyl- and acetyl-CoA. Pleozymes increased total ATP, potentially via flexible enhancement of NAD^+^-dependent catabolic pathways such as glycolysis, fatty acid β-oxidation, and metabolic flux through the TCA cycle. These effects may be favorable for pathologies that compromise metabolism such as brain injury.

## 1. Introduction

Neurological disorders affected 43% of the global population, comprising 3.4 billion people, in 2021 and accounted for 443 million disability-adjusted life years (DALYs), making them the top contributor to the global disease burden [1]. With an aging population, this global burden is expected to increase due to neurodegenerative diseases, particularly Alzheimer’s disease, Parkinson’s disease, and other dementias [2]. Bioenergetics and mitochondrial dysfunction leading to neuronal death have been linked to many neurodegenerative diseases [3].

Central to energy deficiency is NAD^+^, an essential molecule in cellular energy metabolism, regulating numerous metabolic and signaling pathways, including glycolysis, the citric acid cycle, and fatty acid β-oxidation [4,5,6,7,8,9]. Insufficient NAD^+^ adversely affects tissues and organs with high energetic requirements such as the brain and heart, contributing to conditions like aging, infection, metabolic diseases, cardiomyopathy, and neurological disorders [10,11,12]. Supplementation with an NAD^+^ precursor is proposed for increasing intracellular NAD^+^ levels [10,13]. However, solely addressing energy deficiency is often inadequate [14], as cellular dysfunction involves additional complexities such as reactive oxygen species generation and subsequent oxidative stress.

Our group has developed a promising new therapy to address this complex problem. Using a single-step harsh oxidation with nitric acid, we transform coconut shell activated charcoal and modify its structure and chemical properties to create a multifunctional reaction mediator with broad redox potential and therapeutic benefits [15,16,17,18,19,20]. This molecule, named after its substituents as poly(ethylene glycol)-oxidized activated charcoal (PEG-OACs), is readily taken up by cells, co-localizes with mitochondria [21] and exhibits the catalytic activities of multiple mitochondrial and cytoplasmic enzymes. These actions include mimicking superoxide dismutase to neutralize superoxide anion [22], catalyzing the production of NAD^+^ from NADH [21], and oxidizing hydrogen sulfide (H_2_S) to polysulfides [23]. Acknowledging the pleiotropic enzymatic actions, we term PEG-OACs as “pleozymes”, distinguishing them from other synthetic nano-enzymes with highly specific functions [24].

Our group recently discovered that pleozymes increased glycolytic and mitochondrial energy metabolism [18]. Additionally, pleozymes were demonstrated to oxidize NADH to NAD^+^ in solution [21], an essential energy currency for biochemical pathways such as glycolysis, the TCA cycle, and fatty acid β-oxidation [10]. We hypothesized that pleozymes could perform the same reaction inside a living system and oxidize NADH to NAD^+^ in a cell culture model. In this report, intracellular NAD^+^ and NADH were measured, revealing that pleozyme treatment increased total cellular NADH levels while NAD^+^ levels were not significantly elevated. We observed that metabolic flux through certain NAD^+^-driven processes, such as glycolysis and fatty acid β-oxidation were elevated as was total cellular ATP. The results suggested pleozymes increase NAD^+^, subsequently enhance multiple cytoplasmic and mitochondrial NAD^+^-dependent reactions, increasing NAD^+^ consumption, and promoting the accumulation of NADH as a byproduct.

## 2. Methods

### 2.1. Materials

#### 2.1.1. Cell Culture

bEnd.3 murine cortical endothelioma cells (CRL-2299) were acquired from American Type Culture Collection (Manassas, VA, USA). RPMI-1640 Glucose and Glutamine-free Base Media (0500-215C) was acquired from AthenaES (Baltimore, MD, USA). T-75 Culture flasks (10062-860) were acquired from Avantor (Radnor, PA, USA). Phosphate buffered saline (21-040-CV) was acquired from Corning (Corning, NY, USA). Penicillin and Streptomycin Solution (100X) (25-512) was acquired from GenClone (El Cajon, CA, USA). Heat Inactivated Fetal Bovine Serum (A5670801) was acquired from Gibco (Billings, MT, USA). Dulbecco Modified Eagle’s Media (4.5 g/L, no pyruvate) (D5796) was acquired from Sigma-Aldrich (St. Louis, MO, USA). 10 cm Dishes (150464) were acquired from Thermo Fisher Scientific (Waltham, MA, USA)

#### 2.1.2. Cell Assays

NAD/NADH-Glo™ and NADP/NADPH-Glo™ Assay (G9071) and CellTiter-Glo^®^ 2.0 Assay (G9241) were acquired from Promega (Madison, WI, USA). White-walled 384-well plates (781075) were acquired from Greiner Bio-One (Monroe, NC, USA). White-walled 96-well plates (3917) were acquired from Corning (Corning, NY, USA)

#### 2.1.3. Chemical Reagents

Dodecyltetramethylammonium bromide (A10761) and methanol (HPLC Grade) (47192) were acquired from Alfa Aesar (Ward Hill, MA, USA). ^13^C_2_-Glucose (CLM-504-1) and ^13^C_5_-Glutamine (CLM-1166-PK) were acquired from Cambridge Isotope Laboratories (Tewksbury, MA, USA). Ribonucleoside triphosphates (P1132) were acquired from Promega (Madison, WI, USA). Acetonitrile (HPLC grade, 34851), ammonium acetate (09691), ammonium bicarbonate (A6141), isopropanol (HPLC grade, 34863), β-nicotinamide adenine dinucleotide (N8410), β-nicotinamide adenine dinucleotide, reduced disodium salt (N9410), nicotinamide riboside chloride (SMB00907), Trizma base (93352), and Trizma HCl (93363) were acquired from Sigma-Aldrich (St. Louis, MO, USA). Sodium hydroxide (Pellets) (7680) and hydrochloric acid (37%) (BDH3026) was acquired from VWR (Radnor, PA)

#### 2.1.4. Equipment

Thermo Scientific Dionex ICS-6000+ capillary ion chromatography, Thermo IonPac AS11 250 × 2 mm 4 μm column, Thermo Orbitrap Lumos Tribrid mass spectrometer, Thermo Orbitrap IQ-X Tribrid Mass Spectrometer and Thermo Trace Finder software version 5.1 was acquired from Thermo Fisher Scientific (Waltham, MA, USA).

### 2.2. Cell Line

Murine cortical endothelioma (bEnd.3) cells were cultured in T-75 flasks and maintained at 37 °C in 5% CO_2_. Cells were cultured with complete Dulbecco’s modified Eagle’s media (DMEM) containing 4.5 g/mL glucose, 10% heat inactivated fetal bovine serum, and 1% penicillin with streptomycin.

### 2.3. PEG-OACs Synthesis

PEG-OACs (6 h oxidation with fuming nitric acid; Reaction 1) were prepared as published [23]. PEG-OACs subsequently were diluted to 160 µg/mL in sterile PBS, and the final concentration of 4 µg/mL was used in each experiment. This concentration is protective in vitro and in vivo in multiple models of injury [18,21,23,25].

### 2.4. NAD-NADH Glo Luminescent Assay

The bioluminescent NAD/NADH-Glo^TM^ assay was performed as instructed in the protocol with the following adjustments. Cells were treated with pleozymes, NR, or PBS treatment for 15 min, 2 h, or 24 h. Next, cells were incubated with lysis buffer (50 µL 0.2 N NaOH with 2% DTAB/well) for 20 min on a plate shaker at 600 rpm. After 20 min, samples were checked using a microscope to ensure that cells were lysed. Add 50 µL PBS to each well. Mix the plate on the plate shaker briefly. Transfer 40 µL lysate from each well into two Eppendorf tubes, and one tube receives an additional 20 µL 0.4N HCl. Incubate all samples for 30 min at 65 °C. Our lab has an Eppendorf Thermomixer with a 24-tube capacity. Therefore, half of the tubes were incubated in the Eppendorf Thermomixer for the first 15 min at 200 rpm and 65 °C, while the other half were incubated in an aluminum heating bath at 65 °C. After 15 min, the tubes were switched between the Eppendorf Thermomixer and the aluminum heating bath. After neutralizing HCl with 20 µL of 0.5 M Trizma base, the final volume in each Eppendorf tube was 80 µL. The other lysis-containing Eppendorf tube received 40 µL of HCl/Trizma solution (1:1 ratio). Transfer 20 µL from each Eppendorf tube into a white-wall, white-bottom 384-well plate.

Simultaneously, a well of cells without any pleozymes or NR treatment was prepared similarly. Lysate was added to 384-well plate without NAD/NADH-Glo™ Detection Reagent. This well served as a background control for other samples.

Luciferin detection reagent was prepared as instructed in the protocol. Aliquot reconstituted luciferin detection reagent at 1 mL/Eppendorf tube. Aliquot reductase, NAD cycling enzyme, and NAD cycling substrate into Eppendorf tubes. Store the aliquots at −20 °C, protected from light. Stored reductase substrate at −80 °C, protected from light.

Thaw luciferin detection reagent and other reagents at room temperature/4 °C right before use, protected from light. Mixed them and prepared the NAD/NADH-Glo™ detection reagent according to the protocol. Use a multichannel pipet to add 20 µL of the NAD/NADH-Glo™ detection reagent to 384-well plate. Incubate at room temperature for 55 min, protected from light, on a plate orbital shaker. Optimize the luminescence signals using 50% signal from the base-treated well of NR-treated samples. Record signals on a plate reader.

### 2.5. NAD^+^ and NADH Standard Curves in the Glo Luminescent Assay

The powder bottles of NAD^+^ and NADH were equilibrated to room temperature for 1 h before the experiment. A stock of 100 µM NAD^+^ and NADH in 10 mL PBS was made fresh. Subsequently, 10 µM NAD^+^ and NADH in 10 mL PBS, 2 µM NAD^+^ and NADH in 5 mL PBS, and 200 nM NAD^+^ and NADH in 300 µL PBS were made. Next, 150 µL of 200 nM was mixed with 150 µL PBS to achieve 100 nM. Two subsequent concentrations of 50 nM and 25 nM were prepared similarly. Next, 45 µL of each NAD^+^ and NADH concentrations (200 nM, 100 nM, 50 nM, 25 nM, and 0 nM = PBS only) were transferred to a white-wall, white-bottom 96-well plate in duplicate.

To test the effects of pleozymes on NAD^+^ and NADH luminescence, an Eppendorf tube pleozymes 0.4 µg/mL pleozymes in PBS was made. Each 200 nM NAD^+^ or NADH was made by diluting its 2 µM stock in PBS containing 0.4 µg/mL pleozymes. Other concentrations of 200 nM, 100 nM, 50 nM, and 25 nM were made as mentioned above in PBS containing 0.4 µg/mL pleozymes. Transfer 45 µL of each NAD^+^ and NADH concentrations (200 nM, 100 nM, 50 nM, 25 nM, and 0 nM = PBS containing 0.4 µg/mL pleozymes) to a white-wall, white-bottom 96-well plate in duplicate.

Next, 45 µL of the NAD/NADH-Glo™ detection reagent, as prepared above, was added to each well. The plate was incubated at room temperature for 30 min, protected from light. During the first minute of the incubation, the plate was mixed on a plate orbital shaker at 600 rpm. The luminescence signals were optimized using 10% signal from the 200 nM NAD^+^ sample and recorded on a plate reader.

### 2.6. Analysis of Polar ^13^C Metabolites by IC-HRMS

Approximately 80% confluent cells were seeded in 10-cm dishes. Cells were washed with glutamine or glucose-free medium before incubated in fresh medium containing 11.1 mM ^13^C_2_-glucose or 2 mM ^13^C_5_-glutamine for 0, 4, and 24 h. At each time point, the media was either mixed with PBS or pleozymes at a final concentration of 4 µg/mL. After incubation, cells were quickly washed with a pre-measured 6 mL of ice-cold AMBIC wash buffer (0.85% ammonium bicarbonate in water) to remove extra medium components. Wash buffer was removed, and 500 µL of ice-cold extraction buffer was added to each dish. Cells were mechanically lysed using a cell scraper to scrape the solvent and cells to the bottom edge of the dish. Lysate was transferred to a labeled 2 mL Simport tube, flash frozen with liquid nitrogen, and stored at −80 °C.

To determine the incorporation of glucose and glutamine carbon into the glycolysis pathway, intracellular tricarboxylic acid (TCA) cycle, pentose phosphate pathway, and nucleotides, extracts were prepared and analyzed using high-resolution mass spectrometry (HRMS). Metabolites were extracted using cold 80/20 (*v*/*v*) methanol/water with 0.1% ammonium hydroxide. Samples were centrifuged at 17,000× *g* for 5 min at 4 °C, and supernatants were transferred to clean tubes, followed by evaporation to dryness under nitrogen. Samples were reconstituted in 1 mM potassium hydroxide (KOH) in deionized water, then 10 μL was injected into a Thermo Scientific Dionex ICS-6000+ capillary ion chromatography (IC) system containing a Thermo IonPac AS11 250 × 2 mm 4 μm column. The IC flow rate was 360 μL/min (at 30 °C). The gradient conditions are as follows: started with an initial 1 mM KOH, increased to 40 mM at 25 min, then to 100 mM at 39 min, and hold at 100 mM for 10 min. The total run time is 55 min. To assist the desolvation for better sensitivity, methanol was delivered using an external pump and combined with the eluent via a low dead volume mixing tee. Data were acquired using a Thermo Orbitrap IQ-X Tribrid Mass Spectrometer under ESI negative mode at 240 K resolution. Then the raw files were imported to Thermo Trace Finder software for final analysis. The fractional abundance of each isotopolog is calculated by the peak area of the corresponding isotopolog normalized by the sum of all isotopolog areas [26]. The relative abundance of each metabolite was normalized by total peak intensity and to the sample DNA concentration in order to account for differences in cell numbers.

### 2.7. Non-Targeted Metabolomic Profiling

CoAs assay [27] and NADs assay [28] were performed as reported previously by Dr. Philip Lorenzi’s group. Signals obtained from high-resolution mass spectrometry were normalized to the sample DNA concentration.

### 2.8. Analysis of Free Medium- to Long-Chain Fatty Acids by High-Resolution Mass Spectrometry

To determine the relative abundance of free medium- to long-chain fatty acids in cell samples, extracts were prepared and analyzed using high-resolution mass spectrometry (HRMS). Free fatty acids were extracted using ice-cold isopropanol. Samples were vortexed for 10 min, put in −30 °C freezer for 30 min, and then centrifuged at 4000 rpm for 10 min at 4 °C. The supernatants were transferred to clean glass tubes, followed by evaporation to dryness under nitrogen. Samples were reconstituted in 120 μL isopropanol. Then, 10 μL was injected into a Thermo Vanquish liquid chromatography (LC) system containing an Accucore C30 2.1 × 150 mm column with 2.6 µm particle size. Mobile phase A was 10 mM ammonium acetate in 50:50 water:acetonitrile, and mobile phase B was 10 mM ammonium acetate in 5:95 water:acetonitrile. The flow rate was 300 µL/min (at 35 °C), and the gradient conditions were from 5% mobile phase B to 99% B in 20 min and hold at 99% B for 4 min. The total run time was 30 min. Data were acquired using a Thermo Orbitrap Lumos Tribrid mass spectrometer (Thermo Fisher Scientific, Waltham, MA, USA) under ESI negative ionization mode at a resolution of 120,000. Raw files were imported into Skyline-daily (64-bit) 23.1.1.375 software for data analysis. Mass spectrometric abundances were normalized to the sample DNA concentration prior to analysis to account for differences in cell numbers.

### 2.9. Detection of ATP Production in Cells

ATP assay #1: The intracellular production of ATP in bEnd.3 cells was studied by treating the cells with or without 4 μg/mL pleozymes. Cells were passed onto white-bottom, sterile 96-well plates (best compatible with luminescence assay) at 7000 cells/well (n = 6 wells). Cells were treated with pleozymes for 2 and 24 h. ATP detection was accomplished using a luciferin/luciferase luminescence assay following the manufacturer’s instructions (Abcam, Waltham, MA, USA; Luminescent ATP Detection Assay Kit ab113849). Relative luminescence units were converted to ATP concentrations and analyzed between the control and the pleozyme treatment.

ATP assay #2: CellTiter-Glo^®^ 2.0 Assay Promega (Instruction for use of product G9241, G9242, and G4253) was stored at −20 °C and thaw at 4 °C overnight. bEnd.3 cells were seeded on an opaque-walled 96-well plate in DMEM at V = 100 µL/well. The next day, cells were treated with pleozymes for 2 and 24 h. ATP analysis was performed together at one end point. Equilibrate the plate and the reagents to room temperature for 30 min. Add 100 µL of Cell-Titer-Glo^®^ 2.0 Reagent to each well (ratio of reagent: medium was 1:1). The plate was mixed well for 2 min on an orbital shaker to induce cell lysis at 600 rpm. Incubate the plate at room temperature for 10 min to stabilize the luminescence signal, protected from light. Record the luminescence signal, and the integration time was 1 s per well as the guideline.

### 2.10. ATP Standard Curves in Luminescent Assay

High-quality ribonucleoside triphosphates (rATPs, Promega, catalog #P1132) were used as ATP standards. A stock of 10 mM ATP in water at neutral pH was prepared and subsequently diluted in completed DMEM to achieve a series of ATP concentrations, including 1000, 100, 10, 1, and 0.1 nM. The last point, 0 nM, was used to determine background luminescence for all samples and standards points. To test the effect of pleozymes on ATP luminescence, another set of ATP concentrations was made in DMEM containing 0.4 µg/mL pleozymes.

### 2.11. Visualization of Metabolomics Results

For results from ^13^C tracing metabolic flux analyses, isotopic enrichment for each metabolite was calculated using ElemCor [26]. For coenzyme A and fatty acid metabolite analyses, relative mass spectrometric (MS) abundances were normalized relative to sample DNA content and to a ^13^C-malonyl CoA standard (for coenzyme A derivatives only). Mean isotopic enrichment values and normalized relative abundances were plotted as heatmaps in R 4.4.1 (R Foundation for Statistical Computing, Vienna, Austria) using the pheatmap package [29].

### 2.12. Statistical Analysis

Statistical analyses were performed in R 4.4.1 (R Foundation for Statistical Computing, Vienna, Austria), and data were visualized using GraphPad Prism 10.0.0 for Windows (GraphPad Software, Boston, MA, USA). Prior to statistical analysis, groups were assessed for normality using Shapiro–Wilks tests. For unpaired analyses, if normality was satisfied, parametric analyses (Student’s *t*-test for 2 groups, ANOVA for >2 groups) were used. Otherwise, analyses were conducted using nonparametric alternatives (Mann–Whitney U test for 2 groups, Kruskal–Wallis test for >2 groups). Paired analyses were conducted using the Friedman test. All statistical analyses were tested against two-tailed null hypotheses, α = 0.05.

## 3. Results

### 3.1. Pleozymes Increased Cellular NADH Levels

**Figure 1:**
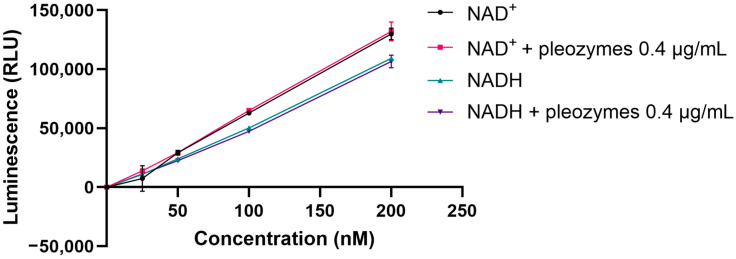
Pleozymes have no effect on NAD^+^- and NADH-associated luminescence. Briefly, 100 µM stocks of NAD^+^ and NADH are prepared in PBS and subsequently diluted in PBS to 200, 100, 50, and 25 nM NAD^+^ and NADH, separately, with and without 0.4 µg/mL pleozymes. The luminescence is recorded following the protocol provided with the NAD^+^/NADH Glo Assay (Promega). N = 1 experiment, each point represents the luminescence mean of two wells and its standard deviation (both directions).

In this study, we investigated the effect of pleozymes on the total cellular levels of NAD^+^ and NADH using a luciferase-coupled luminescence assay. We first established the correlation between relative luminescence units and concentrations of total cellular NAD^+^ and NADH. We found at a theoretical intracellular concentration 0.4 µg/mL (based on 10% uptake of cellular protective concentration of 4 µg/mL), pleozymes had no effect on NAD^+^-associated and NADH-associated luminescence (Figure 1).

**Figure 2:**
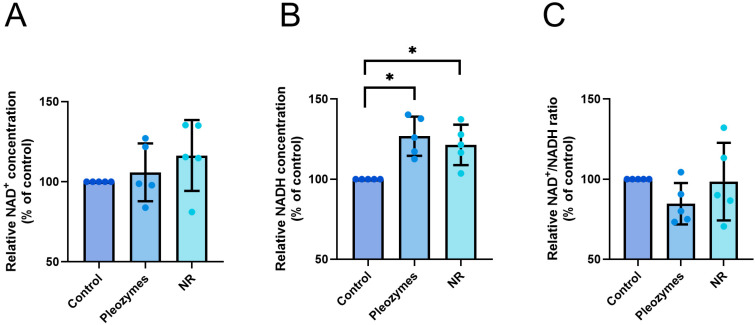
Pleozymes increase intracellular NADH. (**A**) Luminescence assays reveal that pleozymes have no statistical effect on NAD^+^, (**B**) increase NADH, and (**C**) decrease the NAD^+^/NADH ratio. Mean + standard deviation (both directions). N = 5 independent assays. Friedman test. * represents *p* < 0.05.

Cells were treated with nicotinamide riboside (NR) or pleozymes for 24 h, after which luminescence was measured. NR is a cell-permeable precursor for de novo NAD^+^ synthesis; therefore, a positive control for increasing intracellular NAD^+^ levels [10,30,31]. Notably, pleozyme treatment did not affect intracellular NAD^+^ levels but, instead led to an unexpected increase in NADH and a decrease in the NAD^+^/NADH ratio (Figure 2A–C). Treatment with NR resulted in a modest increase in NAD^+^, a significant increase in NADH, and a trend towards decreased NAD^+^/NADH ratios (Figure 2A–C). This was an unexpected outcome, as we anticipated that a NAD^+^ donor would primarily raise the NAD^+^ levels. The results of NR and pleozyme treatments suggest that the production and subsequent cellular consumption of NAD^+^ potentially activates several spontaneous NAD^+^-dependent reactions, generating NADH as a byproduct.

### 3.2. Pleozymes Decrease Isotopic Enrichment of Pyruvate in bEnd.3 Murine Brain Endothelial Cells

**Figure 3:**
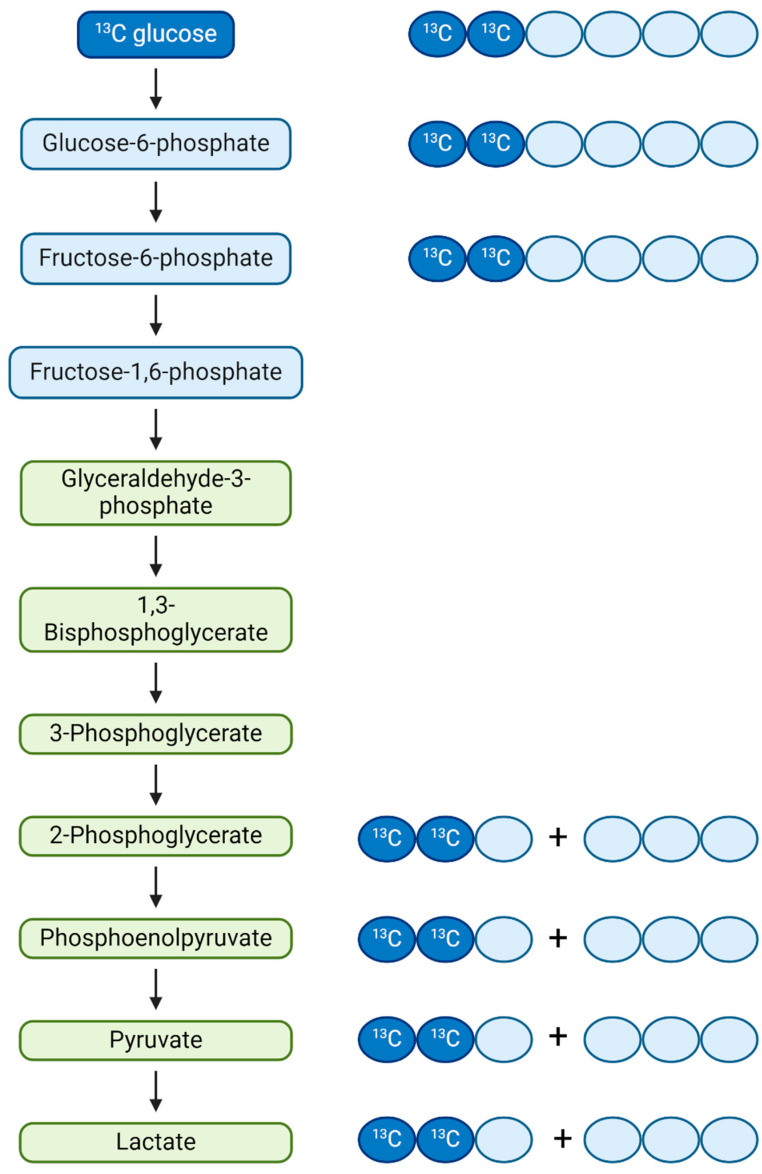
Schematic of ^13^C-glucose isotope tracing metabolic flux analysis. bEnd.3 cells were cultured in media containing 1,2-^13^C-glucose for 24 h. Diagrams indicating isotope-labeled and -unlabeled carbons included for glycolytic intermediates that were analyzed using ion chromatography-mass spectrometry (IC-MS). For steps following the cleavage of fructose-1,6,-bisphosphate to glyceraldehyde-3-phosphate, two 3-carbon molecules are generated per intermediate (highlighted in green), with one containing both ^13^C atoms from the glucose tracer. Figure created using BioRender. (https://BioRender.com/f03b209, accessed on 29 November 2024).

We hypothesized that an increase in the intracellular availability of NAD^+^ would increase the volume of NAD^+^-dependent reactions. To test this hypothesis, we performed a series of metabolomic studies to assess patterns of changes in metabolites. These studies included stable isotope tracing analyses of metabolic fluxes through glycolysis, the pentose phosphate pathway, and the TCA cycle using 1,2-^13^C-glucose as a tracer. Increased generation of a particular metabolite was reported as isotopic enrichment (IE), calculated as a weighted sum of fractional abundances of measured ions (FAMs) for each isotope-labeled ion (i.e., M + 1, M + 2, etc., where “M” represents the molecular weight of the parent ion and “+1”, “+2” indicates the number of ^13^C-labeled carbons) (Figure 3). Each FAM was calculated as a fraction of each labeled ion’s abundance relative to the sum of the abundances of all ions, including the unlabeled parent M + 0 ion. The larger the isotopic enrichment is, the higher the metabolic rate to produce a certain metabolite.

**Figure 4:**
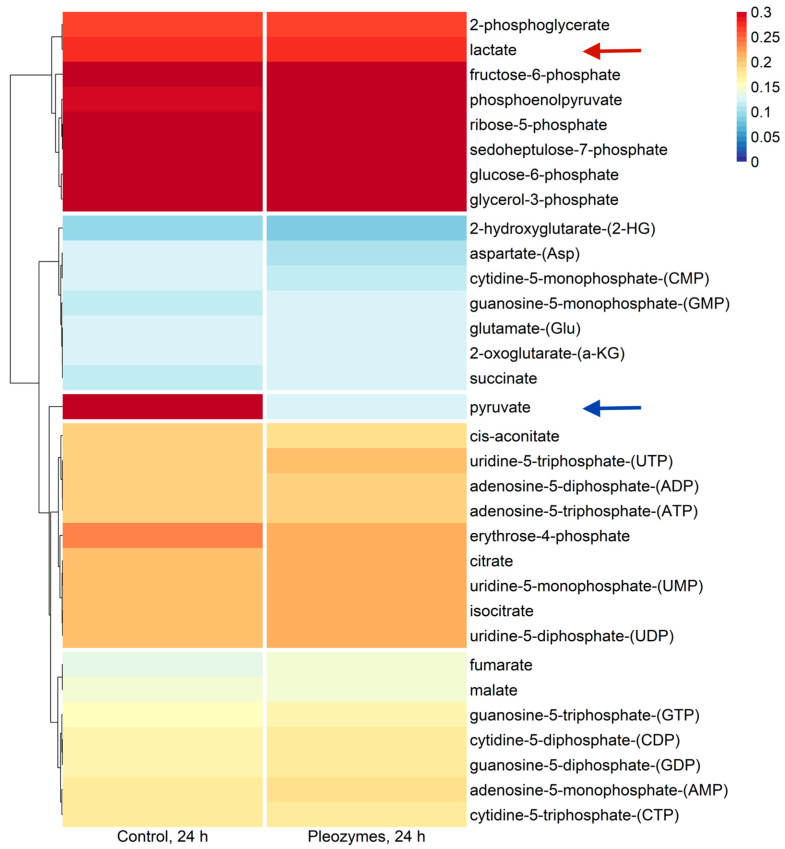
Pleozyme treatment of glycolytically active bEnd.3 murine endothelial cells decreased isotopic enrichment of pyruvate. Columns show metabolite enrichment values from phosphate buffered saline- (control) and pleozyme-treated bEnd.3 cells. Colors represent isotopic enrichment of each metabolite, computed from ^13^C tracing mass spectrometric metabolic flux analysis. Colors are scaled linearly, with higher and lower enrichment values represented by redder and bluer shades, respectively. Basally elevated isotopic enrichment of glycolytic metabolites suggests a higher rate of generation (i.e., increased glycolytic flux). Decreased pyruvate enrichment (blue arrow) in the context of increased glycolytic flux suggests hypothetically increased downstream metabolism of pyruvate with pleozyme treatment. Lactate enrichment (red arrow) remained unaffected with pleozymes. Dendrogram indicates hierarchical clusters of metabolites with similar expression patterns, generated using Ward’s algorithm. N = 1 experiment.

Hierarchical clustering of mean metabolite IEs at baseline (“Control”) and following pleozyme treatment (“Pleozymes”) indicated a distinct clustering of glycolytic and PPP intermediates (Figure 4). Markedly higher IEs indicate a basally elevated glycolytic rate in bEnd.3 cells, which is supported by studies from other groups [32]. The most notable finding of post-pleozyme treatment was a decrease in pyruvate IE (Figure 4). In contrast, lactate IE remained at a similar magnitude of enrichment, suggesting that pleozyme treatment does not block lactate generation. As lactate is exclusively generated from pyruvate in human cells under most conditions [33], we suspected that pleozymes were not blocking pyruvate generation as decreased IE would initially suggest.

**Figure 5:**
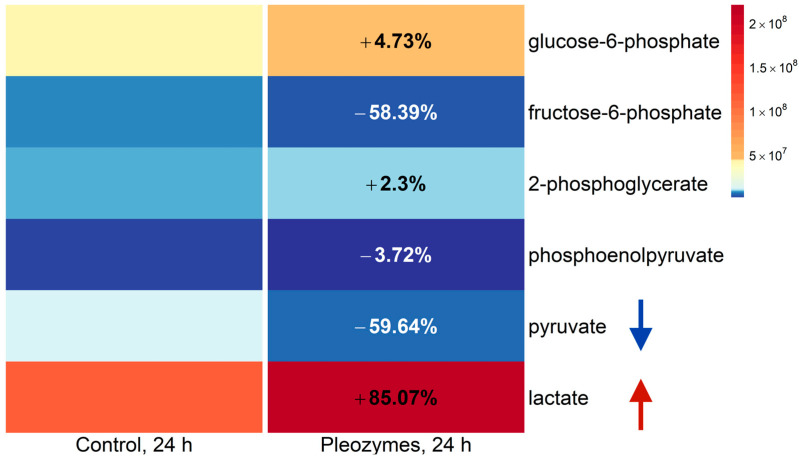
Pleozyme treatment of bEnd.3 cells decreased the intracellular abundance of glycolytically derived pyruvate and increased lactate. Colors represent mean DNA-normalized mass spectrometric abundances of M + 2 isotope-labeled glycolytic intermediates and lactate at baseline (“Control, 24 h”) and following pleozyme treatment. Isotope-labeled intermediates are derived from glycolytic metabolism of 1,2-^13^C-glucose tracer, suggesting that pleozyme treatment hypothetically increased the generation of lactate from glycolytic precursors (blue and red arrows). Decreased fructose-6-phosphate suggests increased metabolism of early glycolytic intermediates toward lactate generation with pleozyme treatment. Colors scaled following quantile binning, with an equal number of data points per bin. N = 1 experiment.

**Figure 6:**
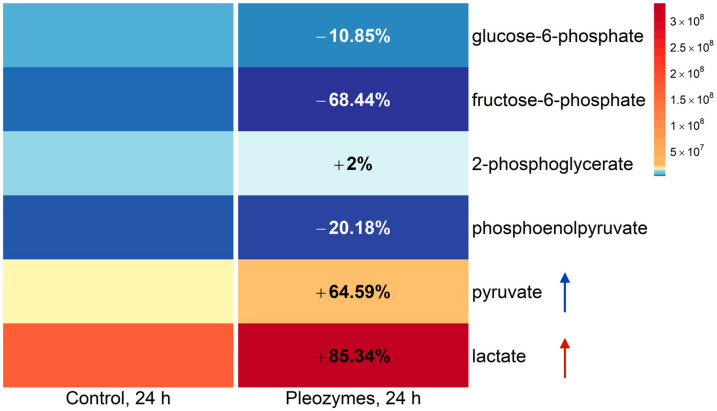
Pleozyme treatment of bEnd.3 cells increased intracellular abundances of non-glycolytically derived pyruvate and lactate. Colors represent mean DNA-normalized mass spectrometric abundances of M + 0 unlabeled glycolytic intermediates and lactate at baseline (“Control, 24 h”) and following pleozyme treatment. Unlabeled intermediates are not derived from glycolytic metabolism of 1,2-^13^C-glucose tracer, suggesting that pleozyme treatment hypothetically increased the generation of lactate from precursors aside from glucose (blue and red arrows). Decreased glucose- and fructose-6-phosphate suggest increased metabolism of residual unlabeled early glycolytic intermediates (present intracellularly prior to tracer glucose treatment) toward lactate generation with pleozyme treatment. Colors scaled following quantile binning, with an equal number of data points per bin. N = 1 experiment.

Decreased isotopic enrichment is a result of decreased FAMs, which may occur if a treatment decreased the abundances of labeled isotopologs relative to the parent M + 0 ion and/or increased the abundance of the M + 0 ion relative to labeled isotopologs. To examine the effect of both possible explanations on pyruvate generation, we analyzed the labeled (M + 2) and parent ion (M + 0) abundances of glycolytic intermediates at baseline and following pleozyme treatment. Pleozyme treatment increased the abundance of isotope-labeled (M + 2) glucose-6-phosphate and lactate, suggesting higher intracellular levels. In comparison, pleozyme treatment decreased the M + 2 abundance of pyruvate, potentially indicating decreased intracellular levels. Increased levels of M + 2 glucose-6-phosphate and lactate with decreased pyruvate suggest that pleozymes increased cell utilization of glucose for glycolysis as well as increased the consumption of glucose-derived pyruvate for lactate generation (Figure 5). Pleozyme treatment also increased the abundance of unlabeled pyruvate and lactate, indicating that pleozymes stimulate pyruvate generation from a glucose-independent pathway (Figure 6). Additionally, pleozyme treatment decreased levels of unlabeled glucose- and fructose-6-phosphate, suggesting increased metabolism of residual intracellular glycolytic intermediates derived from unlabeled glucose prior to cell incubation with isotope-labeled tracer.

**Figure 7:**
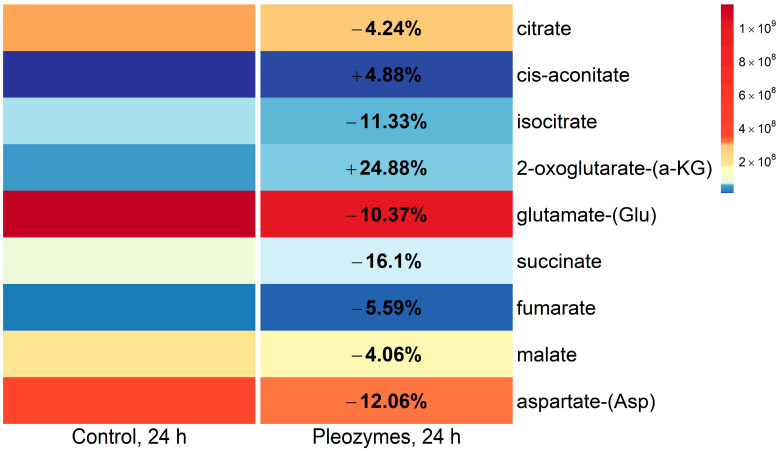
Pleozyme treatment of bEnd.3 cells decreased abundances of TCA cycle intermediates, glutamate, and aspartate, while increasing levels of 2-oxoglutarate. Colors represent mean DNA-normalized mass spectrometric abundances of M + 0 unlabeled TCA cycle intermediates and the amino acids glutamate and aspartate at baseline (“Control, 24 h”) and following pleozyme treatment. A trend, albeit relatively small of decreased TCA metabolite abundances including citrate, isocitrate, succinate, fumarate, and malate may suggest that pleozyme treatment induces the diversion of TCA cycle intermediates toward other biosynthetic pathways. Decreased abundance of glutamate may correspond with an increase abundance of 2-oxoglutarate entering the TCA cycle, which, together with a decreased abundance of aspartate, may indicate increased replenishment of TCA cycle intermediates (anaplerosis) through amino acid transamination. Colors scaled following quantile binning, with an equal number of data points per bin. N = 1 experiment.

One glucose-independent pathway to generating pyruvate involves the oxidation of the TCA cycle intermediate malate to pyruvate by malic enzymes 1 (ME1) and 2 (ME2) [34]. To investigate the possibility that cellular malate is oxidized to pyruvate, we analyzed the unlabeled parent ion abundances of TCA cycle intermediates. Pleozyme treatment of cells effects were quantitatively small, but consistent patterns emerged. Pleozymes decreased the abundances of malate, fumarate, isocitrate, and citrate, consistent with diversion of intermediates away from the TCA cycle for other biosynthetic processes (Figure 7). An increase in 2-oxoglutarate together with decreased glutamate abundances with pleozyme treatment suggests increased TCA anaplerosis (generation of intermediate metabolites) via amino acid transamination, consistent with an increased cellular drive to replenish TCA metabolites.

### 3.3. Pleozyme-Induced Fatty Acid Oxidation

In addition to increasing metabolic flux through glycolysis and the TCA cycle, the increase in NAD^+^ following pleozyme treatment may hypothetically facilitate the NAD^+^-dependent catabolism of fatty acids via β-oxidation, a repetitive process to remove two carbons from the fatty acid chain per cycle. Each oxidative cycle releases one molecule of acetyl-CoA, one NADH, and one FADH_2_ [6,7,35]. Even-chain fatty acids completely break down and release acetyl-CoA, while odd-chain fatty acids (OCFAs) release propionyl-CoA as well, which is further metabolized into succinyl-CoA [6,7,35].

**Figure 8:**
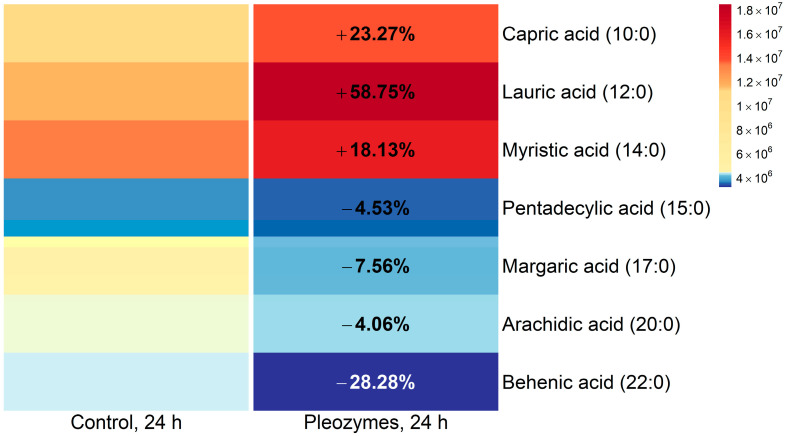
Pleozyme treatment of bEnd.3 cells increased abundances of medium chain fatty acids and myristic acid. Colors represent mean DNA-normalized mass spectrometric intensities of intracellular fatty acid metabolites (N = 3 technical replicates of one experiment). Increased mass spectrometric abundances of capric, lauric, and myristic acids with a concurrent trend toward decreased long- and very long-chain fatty acids (i.e., pentadecylic and behenic acids) suggests hypothetically increased fatty acid oxidation with pleozyme treatment. Colors scaled following quantile binning, with an equal number of data points per bin.

**Figure 9:**
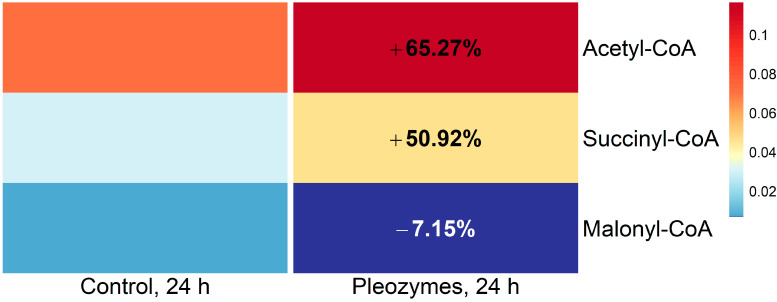
Pleozyme treatment of bEnd.3 cells increased abundances of acetyl- and succinyl-CoA. Colors represent mean DNA-normalized mass spectrometric intensities of intracellular fatty acid metabolites (N = 3 technical replicates of one experiment). Increased mass spectrometric abundances of acetyl- and succinyl-CoA suggests increased higher rate of generation with pleozyme treatment. Pleozymes induce a minor decrease in levels of the fatty acid synthesis precursor malonyl-CoA, suggesting limited metabolic flux through this anabolic pathway. Colors scaled following quantile binning, with an equal number of data points per bin.

Pleozyme treatment showed an increase in the levels of medium-chain and long-chain fatty acids (MCFAs and LCFAs), such as capric acid (C10:0), lauric acid (C12:0), and myristic acid (C14:0). A small reduction in the levels of very long-chain fatty acids (VLCFAs), such as pentadecyclic acid (C15:0), margaric acid (C17:0), arachidic acid (C20:0), and behenic acid (C22:0), was also observed (Figure 8). The results suggested that pleozymes potentially stimulated the breakdown of VLCFAs to LCFAs and subsequently metabolized into MCFAs, generating acetyl-CoA, succinyl-CoA (Figure 9), and NADH (Figure 2) in the process.

Levels of acetyl-CoA and succinyl-CoA increased 65% and 50% with pleozyme treatment, respectively (Figure 9). Notably, pleozymes had a different effect on malonyl-CoA, decreasing levels by 7%. Fatty acid β-oxidation releases acetyl-CoA and succinyl-CoA, while malonyl-CoA inhibits this process and stimulates fatty acid synthesis [6,7,35,36]. These results suggest that pleozymes initiate fatty acid oxidation, leading to the accumulation of the catabolites acetyl-CoA and succinyl-CoA (Figure 9).

### 3.4. Pleozyme Treatment Increased Intracellular ATP Levels

**Figure 10:**
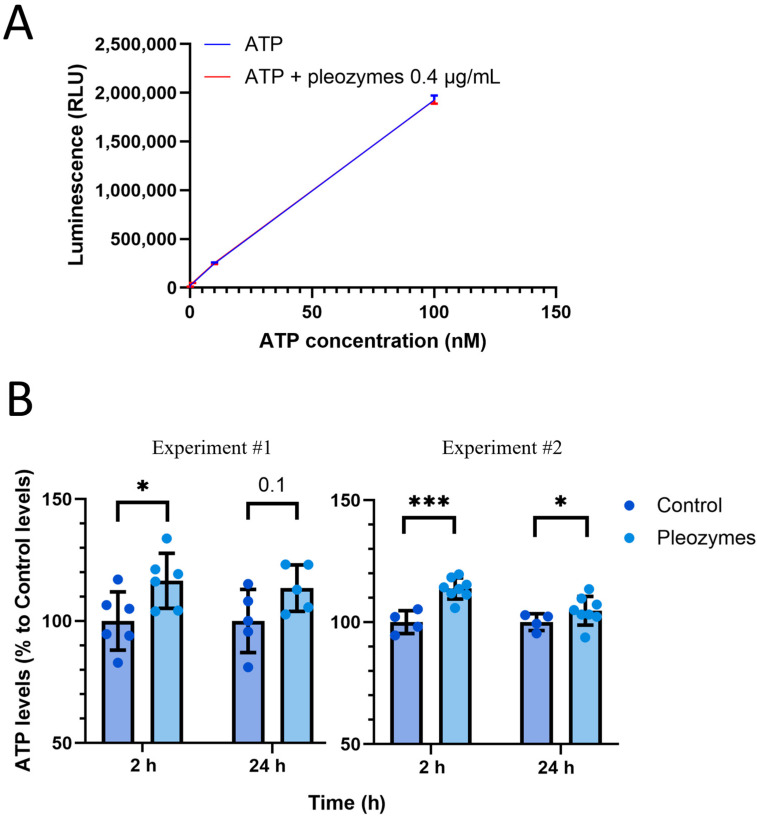
Pleozymes increase intracellular ATP levels after 2 and 24 h of treatment. bEnd.3 murine brain endothelial cells are treated with pleozymes for 2 and 24 h. Then, cell lysate is collected, and ATP levels are measured using a luciferase-coupled luminescence assay. (**A**) Pleozymes at a hypothetical intracellular level of 0.4 µg/mL do not affect ATP-associated luminescence. N = 1 experiment, points represent the mean luminescence of two technical replicates and its standard deviation (ATP in blue, top direction and ATP+pleozymes in red, bottom direction). (**B**) Pleozyme treatment trends toward an increase in intracellular ATP levels at both treatment durations, and this trend is statistically significant at 2 h in two independent assays and at 24 h in one assay. N = 4–8 technical replicates, mean ± standard deviation (both directions), Student’s *t* test, * represents *p* < 0.05, *** represents *p* < 0.001.

Elevated generation of acetyl-CoA and NADH from fatty acid β-oxidation and glycolysis with pleozyme treatment would be expected to increase cellular ATP generation through mitochondrial oxidative phosphorylation. As such, we measured intracellular ATP levels in bEnd.3 cells at baseline and following pleozyme treatment using a luciferase-coupled luminescence assay. The ATP standard curves suggested that pleozymes, at the theoretical intracellular concentration of 0.4 µg/mL, had no effect on the luminescence associated with ATP (Figure 10A).

Cells incubated with pleozymes exhibited significantly higher intracellular ATP levels at 2 h of treatment and a smaller, less consistent trend of higher levels after 24 h of treatment relative to untreated control (Figure 10B). This result is consistent with our previous characterization of the pro-energetic effects of pleozymes in increasing mitochondrial and glycolytic ATP production rates [18]. Together with the preceding observations, this finding suggests that pleozyme-mediated modulation of cellular metabolic pathways may increase cellular energy metabolic capacity.

## 4. Discussion

Reaction 2: Reduction-Oxidation reaction of NADH, resazurin, and PEG-OAC.

NADH + resazurin + PEG-OAC → NAD^+^ + resorufin + PEG-OAC (2)

In a previous study, we found that poly(ethylene glycol) hydrophilic carbon clusters (PEG-HCCs) readily oxidized NADH in a reaction coupled to the reduction of the dye resazurin and cytochrome c [21] (Reaction 2). We hypothesized that this would have metabolic effects on NADH in the mitochondria and found cytoprotective effects involving cyanide poisoning [21]. The precise mechanism by which PEG-HCCs were chemically involved has never been fully understood. It is not possible to determine the effect of poly(ethylene glycol) on PEG-HCC reactivity because HCCs on their own are unstable in buffer and must be functionalized with PEG in order to impart their solubility. Recently, we reported on the effect of PEG on PEG-OACs in a superoxide dismutase (SOD) inhibition assay which measures the SOD activity of a given sample based on how much formazan is reduced by superoxide [25]. We found two different IC_50_ concentrations (OAC: 1.51 µg/mL vs. PEG-OACs 2.64 µg/mL) [25].

We also observed a similar trend in an oxidoreductase-like reaction using NADH and resazurin in conjunction with the OAC or PEG-OAC nanoparticles. In this case, the nanoparticles (4 µg/mL) oxidize NADH to NAD^+^ (2 mM) while also reducing resazurin to resorufin (40 µM), catalyzing the reaction faster than without, as well as in a procedure we described in [21] and (Supplemental Information S1). In an earlier experiment, we found that OACs synthesized from a 2 h oxidation catalyzed the rate of resazurin reduction at a rate of 6.83 × 10^−9^ M/s, meanwhile PEG-OACs synthesized from the same OAC particles catalyzed the reaction at a rate of 7.6 × 10^−10^ M/s, approximately one order of magnitude slower (Figure S1). It is difficult to say for sure if the reason for the change in rates is due to a loss of redox activity caused by changes to the electronic structure of the particle because the carboxylates are now conjugated to PEG, or if steric hindrance due to the poly(ethylene glycol) is the major contributor.

Reaction 3: Pyruvate decarboxylation generates acetyl-CoA and NADH in the mitochondrial matrix [37].

pyruvate + coenzyme A + NAD^+^ → acetyl-CoA + CO_2_ + NADH (3)

The findings from the present study suggest that the oxidation of NADH by pleozymes [21] has biological consequences in that pleozyme treatment increases metabolic flux through NAD^+^-driven metabolic pathways that are directly linked to cellular energy metabolism, including glycolysis, the TCA cycle, and fatty acid β-oxidation. Increased pleozyme-mediated metabolic flux through glycolysis and fatty acid β-oxidation would be expected to increase total cellular NADH levels. We propose that pleozymes increase total cellular NADH by oxidizing mitochondrial NADH to NAD^+^, thus driving forward NAD^+^-dependent reactions that generate NADH. Under circumstances of compromised mitochondrial ATP production, cells compensate by generating ATP through alternative pathways such as lactate and fatty acid oxidation, as long as NAD^+^ is available [6,7,35,38,39]. The depletion of NAD^+^ due to prolonged impairment of oxygen and glucose availability impairs the function and survival of astrocytes during cerebral ischemic injury, while treatment of astrocytes with the redox mediator ginsenoside Rb 1 rescued mitochondrial function by increasing intracellular NAD^+^ levels [40]. Therefore, by oxidizing NADH to NAD^+^ independently from mitochondrial respiratory complex I, pleozymes may ensure that the intracellular pool of NAD^+^ is sufficient for biological functions when mitochondrial function is impaired, thus contributing to cellular resilience against external stressors [18]. Furthermore, the pro-energetic effects of pleozymes may extend to other biologically relevant electron transfer reactions, including reactions catalyzed by iron–sulfur proteins involved in eukaryotic aerobic respiration and flavoprotein function [41]. Our observations thus motivate future examinations of pleozyme-mediated energetic effects in other redox-dependent contexts.

In addition, our findings suggest that pleozymes hypothetically promote cellular energetic flexibility via the generation of pyruvate and lactate from sources other than glucose. The generation of pyruvate from malate decarboxylation occurs under a wide range of physiological circumstances and is a vital link between mitochondrial and cytosolic energy metabolism. The ubiquitously expressed ME1 (cytosolic) and 2 ME (mitochondrial) oxidatively decarboxylate malate, a TCA cycle intermediate, generating cytosolic pyruvate and NADPH [34]. As pleozymes also catalyze the oxidation of NADPH to NADP^+^ [21], it is possible that increased NADP^+^ availability with pleozyme treatment increases the volume of ME-mediated pyruvate generation. Since the carbons comprising malate originate from a wide range of sources apart from glucose via entry into the TCA cycle, increased ME-generated pyruvate with pleozyme treatment would be expected to increase abundances of isotope-unlabeled pyruvate and lactate, as seen here. Also, the increased diversion of malate toward pyruvate generation would be expected to drive forward anaplerotic reactions that replenish TCA cycle intermediates. Malate can be regenerated by aspartate aminotransferase as part of the malate–aspartate shuttle, and has been reported that endothelial cells can flexibly modulate the direction of this shuttle to replenish malate in the TCA cycle in response to metabolic perturbations [42].

**Figure 11:**
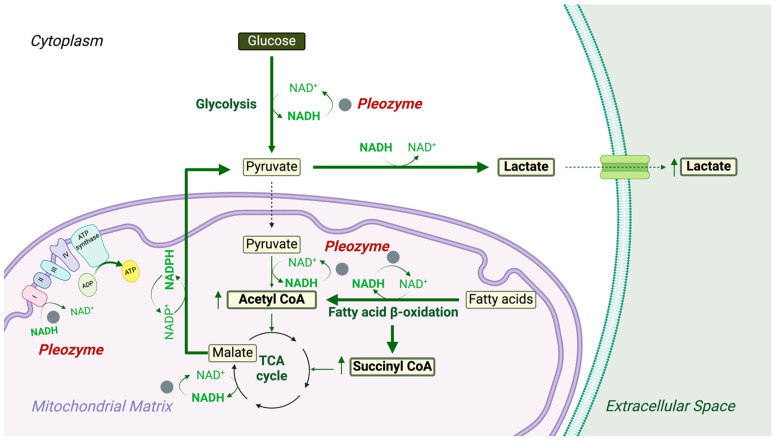
Pleozymes enhance intracellular and mitochondrial energetic pathways and catalytically supply metabolic reactions with NAD^+^ regeneration activities. Localizing in the cytoplasm and mitochondria, pleozymes catalytically accelerate NAD^+^ regeneration from NADH and subsequent ATP production, leading to an increase in the NAD^±^-dependent metabolic rates. Pleozymes accelerate intracellular energy metabolisms through glycolysis and lactate generation. Pleozyme treatment also increases fatty acid β-oxidation and supplies acetyl- and succinyl-CoA to the TCA cycle, driving the cycling reactions and replenishing the lactate precursor, pyruvate, from malate. Figure created using BioRender (https://BioRender.com/d80a234, accessed on 29 November 2024).

Our observation of decreased unlabeled abundances of aspartate and glutamate with pleozyme treatment may thus represent amino acid-dependent replenishment of TCA cycle intermediates to favor increased pyruvate generation. Additionally, increased fatty acid β-oxidation with pleozyme treatment may be another source of unlabeled carbons into the TCA cycle through the generation of acetyl- and succinyl-CoA [6,7]. As mitochondrial NAD^+^ levels regulate the rate of β-oxidation [43], pleozymes may facilitate this catabolic pathway through the oxidation of mitochondrial NADH (Figure 11). Future experiments are needed to elucidate the effects of pleozymes on different anaplerotic pathways and their respective contributions toward cellular energy metabolism and lactate generation.

A result of pleozyme treatment is an increase in NADH levels, which may have clinical benefits. In clinical trials, NADH supplementation in patients with Parkinson’s disease and chronic fatigue syndrome led to improvements in heart rate, reductions in anxiety, and enhanced insulin signaling [44]. By augmenting the cellular capability to produce NAD^+^ from NADH, pleozymes potentially mitochondrial and cellular bioenergetic pathways overall and contribute toward enhanced metabolic flexibility, with their pleotropic actions addressing any subsequent reactive oxygen or sulfur species generation.

## 5. Conclusions

Pleozymes demonstrate therapeutic potential by enhancing metabolic flexibility and augmenting energy production, thereby addressing critical cellular defense mechanisms following injury or in the context of metabolic disorders. Future investigations should focus on the direct quantification of pyruvate and key energy metabolites, including NAD^+^, NADH, and ATP, particularly in an energy-deficient model, to further validate the pleozyme’s efficacy.

## Figures and Tables

**Reaction 1:**
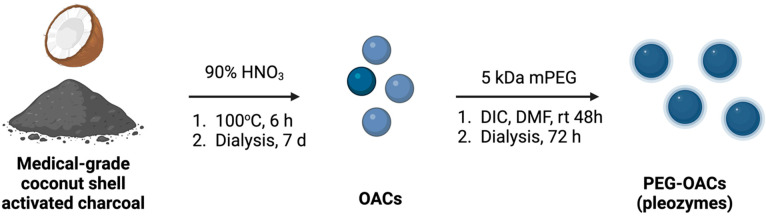
Schematic of PEG-OAC (pleozyme) synthesis. The synthesis starts by oxidizing a medicinal ingredient, coconut shell activated charcoal, with fuming nitric acid (90% HNO_3_) at 100 °C for 6 h in a round-bottom flash. The products are subsequently purified using water bath dialysis for 7 days and filtered through a 0.22 μm membrane to collect oxidized activated charcoal (OACs). Next, OACs are left to react with 5 kDa polyethylene glycol (PEG) in DIC and DMF for 48 h at room temperature. Subsequently, bath dialysis and filtration are performed to produce sterile PEG-OACs (pleozymes). Figure created using BioRender.

## Data Availability

The raw data supporting the conclusions of this article will be made available by the authors on request.

## Supplementary Materials

The following supporting information can be downloaded at: https://www.mdpi.com/article/10.3390/nano14242017/s1, Figure S1. Effect of PEGylation on OAC-catalyzed NADH-resazurin oxidoreduction. Preliminary data shows that OACs are notably faster without PEGylation.
