# Supplementary material for: Pleozymes: Pleiotropic Oxidized Carbon Nanozymes Enhance Cellular Metabolic Flexibility
Source: Nanomaterials (Basel). 2024 Dec 15;14(24):2017. doi: 10.3390/nano14242017 (PMC11728746; doi:10.3390/nano14242017)
Supplement: Supplementary file 1 [file nanomaterials-14-02017-s001.zip › nanomaterials-3331140-supplementary.pdf]

Supplemental Information

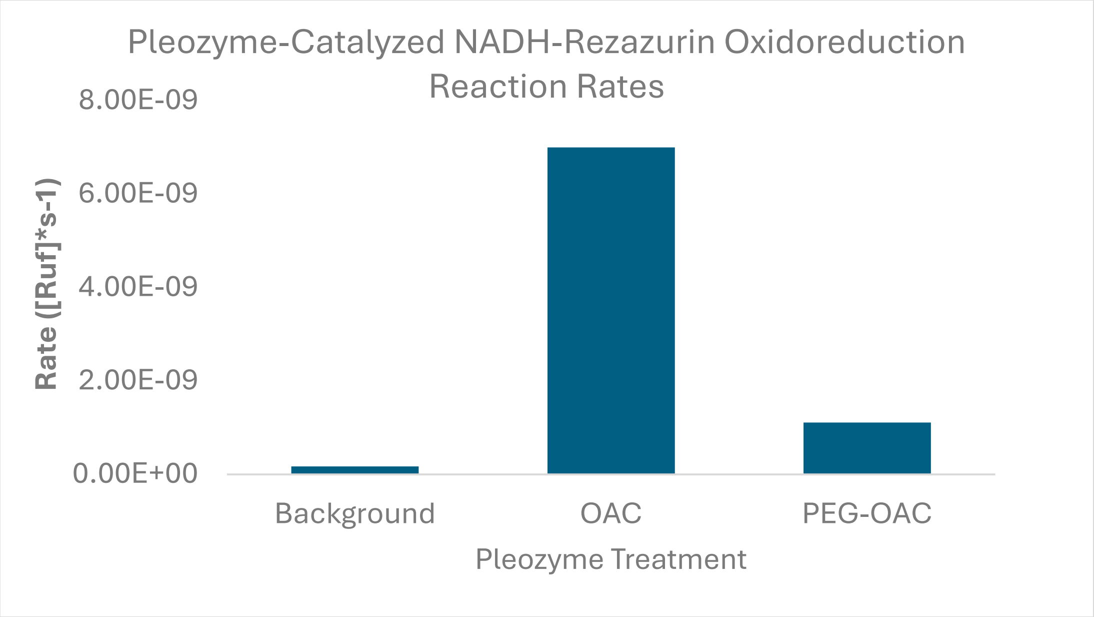

Figure S1. Effect of PEGylation on OAC-catalyzed NADH-resazurin oxidoreduction. Preliminary data shows that OACs are notably faster without PEGylation.

<u>Procedure</u>

Oxidized activated charcoal and poly(ethylene glycol)-functionalized oxidized activated charcoal were synthesized using a 2 hour oxidation step according to McHugh et al.[1] After preparation, a nanoparticle catalysis experiment was performed using NADH (2 mM) as the reductant, and resazurin (40 uM) as the oxidizer in PBS with PEG-OACs or OACs (4 ug/mL), or PBS alone. The rate of reaction was monitored using an Agilent 8453 UV-Vis spectrophotometer operating in kinetics mode (sample rate: 2/min; duration 10 min) tracking 570 nm and 600 nm wavelengths which correspond to the peaks of both resazurin and the reduction product resorufin and described in Derry et al.[2]

The rate was calculated by knowing the concentration of resorufin in the reaction mixture at each time point and applying a linear regression to obtain the slope. The concentration was calculated using the formula provided by ThermoFisher and reproduced here in part[3]:

$$[Ruf] = \frac{(\varepsilon_{ox})\lambda_2 A\lambda_1 - (\varepsilon_{ox})\lambda_1 A\lambda_2}{(\varepsilon_{red})\lambda_1(\varepsilon_{ox})\lambda_2 - (\varepsilon_{ox})\lambda_1(\varepsilon_{red})\lambda_2}$$

The extinction coefficients are provided by ThermoFisher and are reproduced here:

	Resazurin (ε_{ox})	Resorufin (ε_{red})
570 nm ($A\lambda_1$)	80,586	117,216
600 nm ($A\lambda_2$)	155,677	14,652

References

1. McHugh, E.A.; Liopo, A.V.; Mendoza, K.; Robertson, C.S.; Wu, G.; Wang, Z.; Chen, W.; Beckham, J.L.; Derry, P.J.; Kent, T.A.; et al. Oxidized Activated Charcoal Nanozymes: Synthesis, and Optimization for In Vitro and In Vivo Bioactivity for Traumatic Brain Injury. *Adv Mater* **2023**, e2211239, doi:10.1002/adma.202211239.

2. Derry, P.J.; Nilewski, L.G.; Sikkema, W.K.A.; Mendoza, K.; Jalilov, A.; Berka, V.; McHugh, E.A.; Tsai, A.L.; Tour, J.M.; Kent, T.A. Catalytic oxidation and reduction reactions of hydrophilic carbon clusters with NADH and cytochrome C: features of an electron transport nanozyme. *Nanoscale* **2019**, *11*, 10791-10807, doi:10.1039/c9nr00807a.

3. AlamarBlue Assay. Rev. 1.0. ThermoFisher. [Online] Available: https://tools.thermofisher.com/content/sfs/manuals/PI-DAL1025-1100_TI%20alamarBlue%20Rev%201.1.pdf
